# Risk Factors and Outcomes of Choroidal Neovascularization Secondary to Central Serous Chorioretinopathy

**DOI:** 10.1038/s41598-019-40406-y

**Published:** 2019-03-08

**Authors:** Ga-In Lee, A. Young Kim, Se Woong Kang, Soo Chang Cho, Kyu Hyung Park, Sang Jin Kim, Kyung Tae Kim

**Affiliations:** 1 Department of Ophthalmology, Samsung Medical Center, Sungkyunkwan University School of Medicine, Seoul, Republic of Korea; 2 Department of Ophthalmology, Seoul National University College of Medicine, Seoul National University Bundang Hospital, Seongnam, Republic of Korea; 3 Department of Ophthalmology, Kyungpook National University Hospital, Daegu, Republic of Korea

## Abstract

We identified clinical characteristics and risk factors of choroidal neovascularization (CNV) in eyes with prior episode of central serous chorioretinopathy (CSC). This retrospective case-control study included those initially diagnosed with CSC and developed CNV secondarily (Group 1, n = 16), those diagnosed with CNV in eyes of previous putative CSC (Group 2, n = 14), and those initially diagnosed with CSC, and did not develop CNV secondarily, as a control group for Group 1 (Group 3, n = 250). Clinical characteristics including treatment outcomes were assessed. Demographics and multimodal imaging at the time of CSC diagnosis of secondary CNV were compared between the groups to identify risk factors. Duration from diagnosis of CSC to development of CNV in Group 1 was 40.2 ± 42.0 months. Classic CNV was noted in 23 (76.7%) eyes. After treatment with intravitreal antiangiogenics with average of 4.9 times, visual acuity improved in Group 1 and Group 2 (*p* = 0.002). Multivariate analysis revealed that systemic hypertension, pigmentary changes, and double layer sign were associated with development of CNV secondary to CSC (*p* < 0.05). Hypertension, pigmentary changes, and double layer sign were independent risk factors for CNV secondary to CSC. The CNV’s responded well to treatment, resulting in improved vision.

## Introduction

Choroidal neovascularization (CNV) is an important complication of chronic central serous chorioretinopathy (CSC). Development of CNV is one of the major causes of reduced vision seen during long-term follow-up of patients with CSC. The prevalence of CNV secondary to CSC ranges from 2–15.6 percent^1,2^. The detection of CNV in patients with CSC can be more challenging than the diagnosis of idiopathic CNV, because of the diffuse decompensation and abnormalities of the retinal pigment epithelium (RPE) layer seen in CSC^3,4^. Consequently, the use of multimodal imaging, including color fundus photography, optical coherence tomography (OCT), dye based angiography, and recently, OCT angiography for detection of CNV secondary to CSC has been reported^5–7^. A large number of cases with co-existing features of CSC and CNV are thought to be pachychoroid neovasculopathy, as reported in Freund *et al*.^8–10^. That is, they presented the cases in which type 1 CNV under RPE undulation (double layer sign) was simultaneously present in the eyes with pachychoroid features, such as choroidal vascular hyperpermeabililty, increased choroidal thickness, and pachyvessels. Indeed, the frequency of these conditions is much higher than what was previously thought. However, these cases should be distinguished from cases with secondary CNV, where a diagnosis of CSC had been confirmed. Pathogenesis of secondary CNV is currently unclear. It is well known that it might occur after laser photocoagulation or photodynamic therapy (PDT) for CSC^11–13^. However, few studies have elucidated the potential risk factors for secondary CNV development in eyes after a prior episode of CSC. Furthermore, although verteporfin PDT^14,15^ and anti-vascular endothelial growth factor (VEGF) therapy^16–19^ have been reported to be effective for CNV secondary to CSC, the outcomes of such treatment have not yet been sufficiently addressed through studies of large case numbers. The purpose of this study was to determine the risk factors for development of CNV secondary to CSC and to report functional and anatomical outcomes of the treatment for the CNV.

## Results

### Characteristics of Patients with CNV Secondary to CSC

Demographic characteristics of patients in Group 1 and Group 2 are summarized in Table 1. Secondary CNV characteristics of Group 1 and Group 2 are shown in Table 2. On fluorescein angiography (FA), classic type CNV was revealed in 23 (76.7%) eyes while occult type CNV was detected in 7 (23.3%) eyes. Predominance of classic type CNV compared to occult type CNV was statistically significant (*p* = 0.003). Fourteen (46.7%) eyes revealed subfoveal location. Thirteen (43.3%) eyes revealed juxtafoveal location while 3 (10.0%) eyes had extrafoveal location. The number of eyes with extrafoveal location was significantly (*p* < 0.0001) less than that with subfoveal or juxtafoveal location. Subretinal hemorrhage developed in 17 (56.7%) eyes. Hyperpermeability on early phase of indocyanine green angiography (ICGA) was detected in 25 (83.3%) eyes. Subretinal turbid exudation occurred in 24 (80.0%) eyes.

**Table 1:** Patient demographics of Group 1 & 2.

	Group 1 & 2 (N = 30)	Group 1 (N = 16)	Group 2 (N = 14)	P-value
Age at diagnosis of CNV (years) (range)	50.6 ± 10.1 (30–74)	51.7 ± 8.2 (39–67)	49.3 ± 12.2 (30–74)	0.61*
Gender (male: female)	23: 7 (3.3: 1)	11:5 (2.2:1)	12:2 (6:1)	0.26^†^
Mean duration of follow-up (months) (range)	60.0 ± 53.4 (2.2–145.2)	77.3 ± 54.7 (9.3–145.2)	37.0 ± 42.0 (2.2–135.9)	0.02*
Systemic disease				
Diabetes mellitus, n (%)	4 (13.3)	2 (12.5)	2 (14.3)	0.65^†^
Hypertension, n (%)	10 (33.3)	7 (43.8)	3 (21.4)	0.18^†^
Treated eye (Right: Left)	13:17	5:11	8:6	0.15^†^
Duration from diagnosis of CSC to development of secondary CNV in Group 1 (months) (median) [range]	N. A.	50.6 ± 50.3 (40.5) [0.6–156.0]	N. A.	
LogMAR BCVA at diagnosis of CNV	0.54 ± 0.50	0.48 ± 0.52	0.61 ± 0.49	0.28*

CNV = choroidal neovascularization; BCVA = best corrected visual acuity; N. A. = not applicable; logMAR = logarithm of minimal angle of resolution.

Group 1: Patients with secondary CNV in eyes with previous CSC.

Group 2: Patients diagnosed with CNV in eyes with putative chronic CSC beforehand.

*Statistical analysis with Mann-Whitney *U* tests between Group 1 & 2.

^†^Statistical analysis with Fisher’s exact tests between Group 1 & 2.

**Table 2:** CNV characteristics of Group 1 & 2.

Characteristics	Patients (N = 30)	P-value
CNV type		
Classic, n (%)	23 (76.7)	0.003*
Occult, n (%)	7 (23.3)	
CNV location		
Subfoveal, n (%)	14 (46.7)	
Juxtafoveal, n (%)	13 (43.3)	
Extrafoveal, n (%)	3 (10.0)	0.0001*
Subretinal hemorrhage, n (%)	17 (56.7)	
Hyperpermeability on ICGA, n (%)	25 (83.3)	
Subretinal turbid exudation, n (%)	24 (80.0)	

ICGA = indocyanine green angiography.

Group 1: Patients with secondary CNV in eyes with previous CSC.

Group 2: Patients diagnosed with CNV in eyes with putative chronic CSC beforehand.

*Statistical analysis with Chi-square tests.

### Treatment outcomes of CNV secondary to CSC

In Group 1, CSC treatment consisted of PDT in 8 (50.0%) patients, focal laser in 2 (12.5%) patients, combination therapy of PDT and focal laser in 2 (12.5%) patients, and carbonic anhydrase inhibitor medication in 5 (31.3%) patients. Three (18.8%) patients were observed without treatment. CNV treatment methods included anti-VEGF injection and PDT for Group 1 and Group 2 (Table 3). One patient underwent pars plana vitrectomy and removal of subretinal membrane. One patient was observed without treatment. Of all patients, 14 (46.7%) underwent intravitreal bevacizumab injection, 5 (16.7%) underwent intravitreal ranibizumab injection, and one (3.3%) underwent intravitreal aflibercept injection. The number of patients that underwent intravitreal anti-VEGF injection was 22 (73.3%) with including 2 patients those who have received a combined injection. The average number of injection was 4.9. One (3.3%) patient underwent PDT. Five (16.7%) patients underwent combination therapy consisting of PDT and anti -VEGF injection.

**Table 3:** CNV Treatment Methods in Group 1 & 2.

Methods	Patients (N = 30)
Intravitreal anti-VEGF injection, n (%) (mean)	22 (73.3) (4.9)
Bevacizumab, n	14
Ranibizumab, n	5
Aflibercept, n	1
Bevacizumab + Ranibizumab, n	1
Bevacizumab + Ranibizumab + Aflibercept, n	1
PDT + intravitreal anti-VEGF injection, n (%)	5 (16.7)
Bevacizumab + PDT, n	3
Bevacizumab + Ranibizumab + PDT, n	2
PDT, n (%)	1 (3.3)
Surgery*, n (%)	1 (3.3)
No treatment, n (%)	1 (3.3)

VEGF = vascular endothelial growth factor; PDT = photodynamic therapy.

Group 1: Patients with secondary CNV in eyes with previous CSC.

Group 2: Patients diagnosed with CNV in eyes with putative chronic CSC beforehand.

*Pars plana vitrectomy, removal of subretinal membrane.

**Figure 1:**
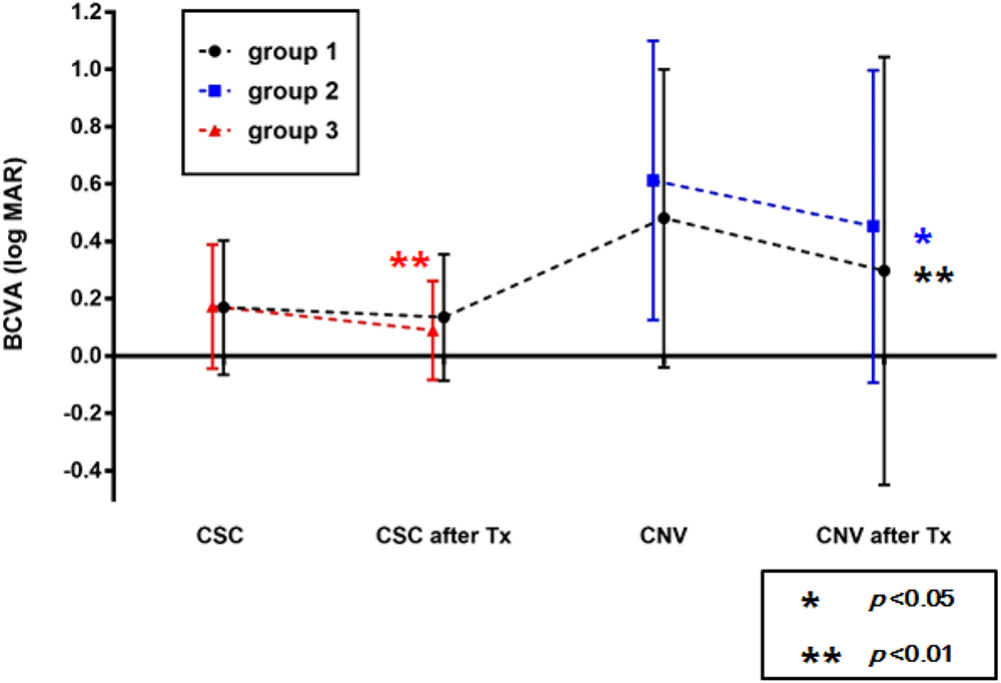
The serial changes of best-corrected visual acuity (BCVA) in logMAR scale between each group from diagnosis to last follow-up. BCVA was significantly improved when compared with pretreatment values of CNV in group 1(*p* = 0.003) and group 2 (*p* = 0.04) and that of CSC in group3 (*p* < 0.001).

**Figure 2:**
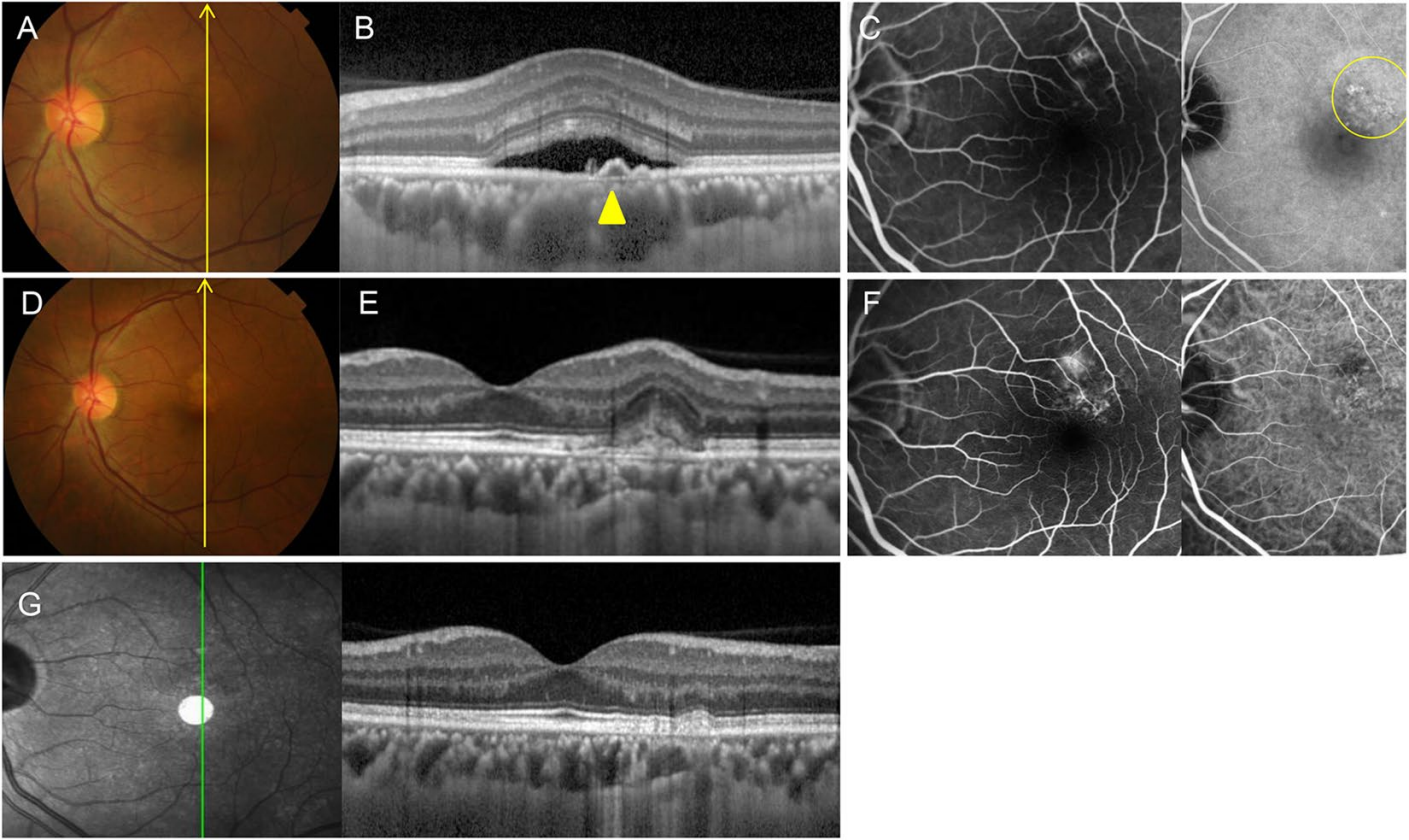
Group 1: Central serous chorioretinopathy in a 47-year-old male first presented in 2013. (**A**) Fundus color photography shows pigmentary change and serous elevation. (**B**) Optical coherence tomography (OCT) with enhanced depth imaging vertical section shows a subretinal fluid with double layer sign (yellow arrowhead) and large choroidal vessels. (**C**) Late phase of fluorescein angiography (FA) and indocyanine green angiography (ICGA) shows a leaking point and hyperfluorescence spots (region in the circle of yellow line) on perifoveal area. CSC was treated with photodynamic therapy (PDT). (**D**) In 2016, fundus color photography shows RPE changes. (**E**) OCT scan shows a CNV membrane and disruption of RPE layer. (**F**) FA and ICGA shows an early hyperfluorescence with late leakage. The patient was treated with three intravitreal bevacizumab injections. (**G**) OCT scan shows significant improvement without subretinal fluid or CNV membrane. BCVA was 20/20 without further treatment.

**Figure 3:**
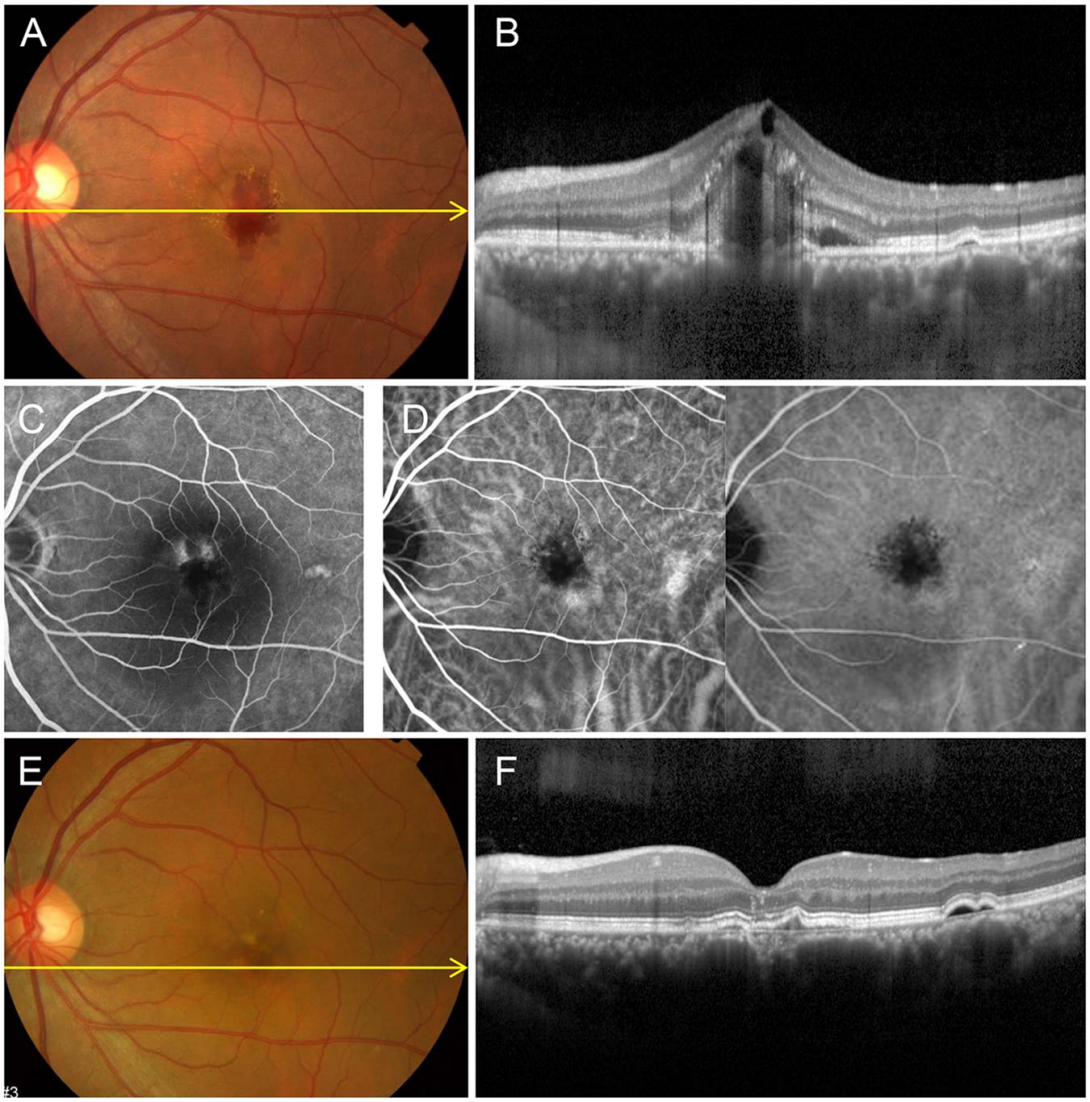
Group 2: Choroidal neovascularization (CNV) in a 48-year-old male first presented in 2009 with a history of central serous chorioretinopathy (CSC) 10 years ago. (**A**) Fundus color photography shows subretinal hemorrhage and exudation. (**B**) Optical coherence tomography (OCT) with enhanced depth imaging horizontal section shows RPE disruption, hemorrhage, cystoid macula edema and pachychoroid. (**C**) Late phase of fluorescein angiography shows two leaking points and hyperfluorescence on perifoveal area. (**D**) Indocyanine green angiography shows choroidal hyperpermeability in early phase and blocked hypofluorescence with leakage in late phase. CNV was treated with three intravitreal bevacizumab injections. (**E**) After treatment, fundus color photography shows RPE changes. (**F**) OCT scan shows significant improvement without subretinal exudation or CNV membrane except double layer sign and RPE changes. BCVA was 20/20 without further treatment.

**Figure 4:**
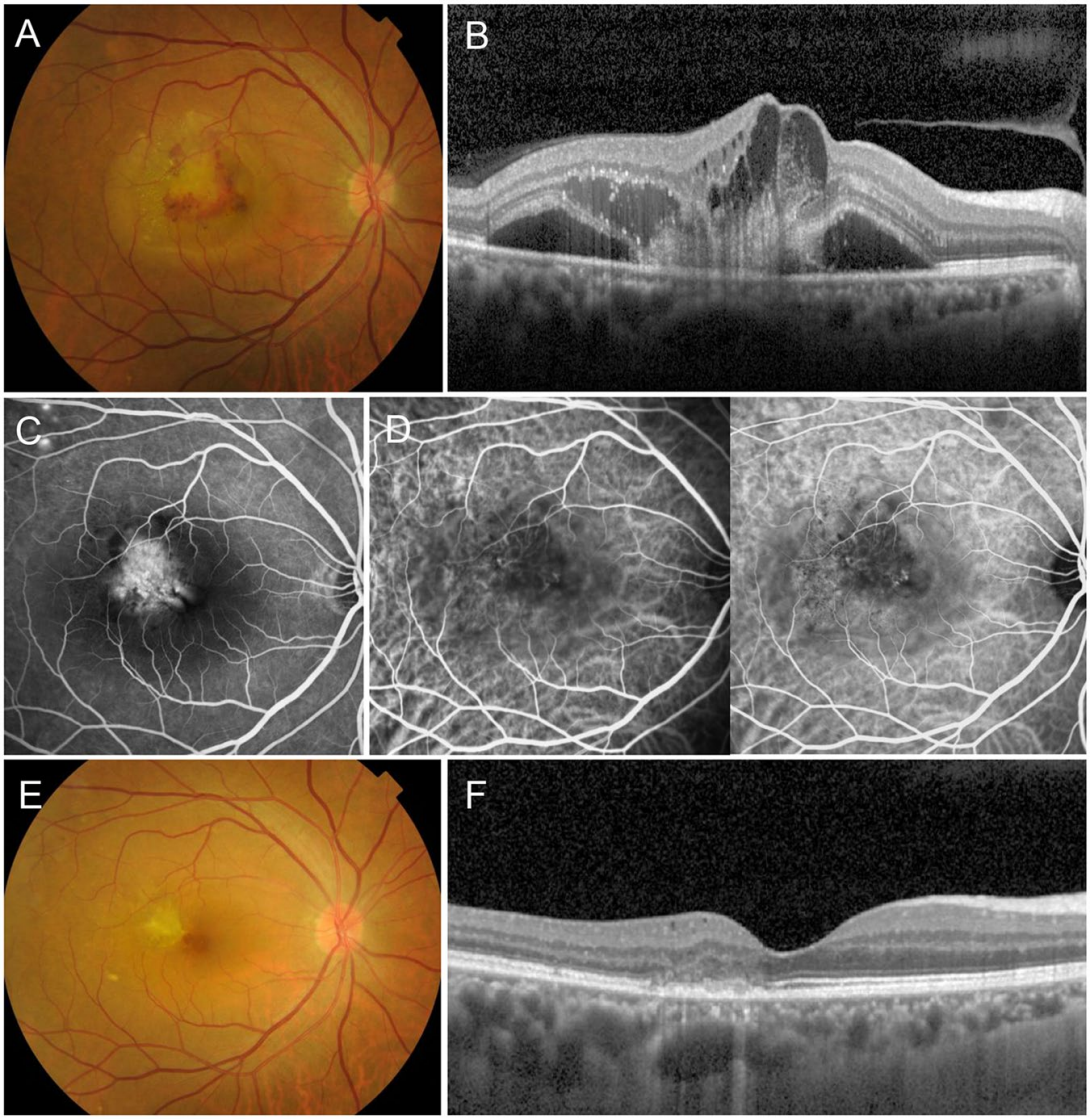
Group 2: Choroidal neovascularization (CNV) in a 60-year-old male first presented in 2013. Fundus color photography shows subretinal hemorrhage and turbid exudation (**A**). Optical coherence tomography (OCT) horizontal section shows retinal pigment epithelial (RPE) disruption, hemorrhage, subretinal exudation, cystoid macula edema and pachychoroid (**B**). Fluorescein angiography shows intense hyperfluorescence with leakage suspicious for a classic CNV, along with focal punctate hyperfluorecsence in the superior temporal arcade. (**C**). Indocyanine green angiography shows mild choroidal hyperpermeability and neovascular structure (**D**). CNV was treated with five intravitreal ranibizumab injections. After treatment, fundus color photography shows inactive CNV membrane (**E**). OCT scan shows significant improvement without subretinal exudation except RPE disruption (**F**). The vision has been improved to 20/25 without further treatment and the patient has been in remission state for a 6-year follow-up period without a recurrence.

CNV treatment outcomes in Group 1 and Group 2 are summarized in Table 4. Twenty-two (73.3%) patients achieved anatomically stable state after treatment, and manifested drying of fluid and consolidation of CNV with regression on OCT (*p* = 0.01). They were observed with regular follow-up. Of 30 eyes, 6 (20.0%) eyes remained subretinal fluid or subretinal membrane stationary after treatment. Two patients did not visit the clinic after the third intravitreal injection of bevacizumab. Visual acuity significantly improved from 0.54 ± 0.50 at diagnosis of CNV to 0.35 ± 0.65 at the last visit (*p* = 0.002). We revealed the changes of visual acuity in each group from diagnosis to last follow-up in Fig. 1. Representative cases in each group are presented in Figs 2, 3 and 4.

**Table 4:** Secondary CNV treatment outcomes with stable state in Group 1 & 2.

Outcomes	Patients (N = 30)	P-value
Anatomical outcome		0.01**
Stable state* after treatment and observation with regular follow-up, n (%)	22 (73.3)	
Subretinal fluid or subretinal membrane stationary after treatment, n (%)	6 (20.0)	
Follow-up loss, n (%)	2 (6.7)	
Duration until CNV regression^†^ (median) [range]	10.4 ± 16.5 (5.2) [1.2–75.8]	
Visual outcome		0.002^‡^
LogMAR BCVA at diagnosis of CNV	0.54 ± 0.50	
LogMAR BCVA at last visit	0.35 ± 0.65	

Group 1: Patients with secondary CNV in eyes with previous CSC.

Group 2: Patients diagnosed with CNV in eyes with putative chronic CSC beforehand.

*Stable state = CNV regression state with no further treatment.

^†^CNV regression = consolidation or complete disappearance of subretinal fluid in OCT images.

**Statistical analysis with Chi-square tests.

^‡^Statistical analysis with Wilcoxon signed rank tests.

### Risk factors of CNV secondary to CSC

Risk factors of secondary CNV subsequent to CSC were analyzed using univariate logistic regression analysis of baseline characteristics of Group 1 and Group 3 (Supplement Table 1). Systemic hypertension was noted in 43.8% of CSC patients with secondary CNV involvement (Group 1) and in 19.2% of CSC patients without secondary CNV involvement (Group 3) (*p* = 0.03). Macula edema was revealed in 6.3% of Group 1 and 0.4% of Group 3 (*p* = 0.046). Ellipsoid zone disruption was presented in 31.2% of Group 1 and 9.6% of Group 3 (*p* = 0.008). Double layer sign of RPE/Bruch’s membrane complex was observed in 50.0% of Group 1 and 9.6% of Group 3 (*p* = 0.007). There was significant (*p* = 0.01) difference in the number of leaking points between Group 1 and Group 3. RPE change was presented in 75.0% of Group 1 and 38.8% of Group 3 (*p* = 0.004).

Relationship between risk factors was examined using multivariate logistic regression analysis. Hypertension (OR, 7.72; 95% confidence interval [CI], 1.26–47.36; *p* = 0.03), double layer sign (OR, 12.32; 95% CI, 2.04–74.4; *p* = 0.006) and RPE change in fundus color photography (OR, 9.00; 95% CI, 1.46–55.62; *p* = 0.02) remained significant risk factors after adjusting for other factors. There was no significant difference between Group 1 and Group 3 in the following factors: diabetes mellitus, age, sex, focal laser, photodynamic therapy, sub-retinal deposits, drusen, drusen-like deposits, macula edema, pigment epithelial detachment (PED), RPE tear, subretinal fibrin, ellipsoid disruption, location and number of hyperfluorescent spots, hyperpermeability on ICGA, pachyvessels on OCT, leakage pattern, leaking point, or descending atrophic tract (Table 5).

**Table 5:** Risk factors of secondary CNV subsequent to development of CSC with multivariate regression analysis.

Parameters	OR (95% CI)	P-value*
Age	0.92 (0.83–1.02)	0.09
Systemic hypertension	**7.72 (1.26–47.36)**	**0.03**
Macula edema	0.48 (0.005–44.55)	0.75
Double layer sign	**12.32 (2.04–74.4)**	**0.006**
Ellipsoid zone disruption	2.75 (0.47–16.14)	0.26
Hyperfluorescent spots-perifoveal area	0.08 (0.02–0.42)	0.003
Leaking points	2.02 (0.60–6.82)	0.26
RPE changes	**9.00 (1.46–55.62)**	**0.02**

RPE = retinal pigment epithelium; CI = confidence interval; OR = odds ratio.

*Values in bold are statistically significant results.

## Discussion

We characterized the CSC that developed CNV afterwards. Treatment outcomes of secondary CNV were analyzed. This study indicated that systemic hypertension, presence of double layer sign, and RPE changes were independent risk factors of secondary CNV subsequent to CSC. CNVs were most frequently the classic type in subfoveal and juxtafoveal location. Most of the patients with secondary CNV revealed excellent visual and anatomical improvement with anti-VEGF treatment.

Occurrence of secondary CNV subsequent to CSC has been reported by several authors^18,20^. Secondary CNV could develop as a complication of photodynamic therapy for CSC. Choroidal vascular remodeling and decreased choroidal permeability after PDT constitute the rationale of this therapy for CSC^21^. However, CNV could develop after PDT for CSC because of its pro-inflammatory effect^12^. Additionally, PDT may result in significant reduction in chorioretinal perfusion that in turn can increase risk of secondary CNV. That is, localized ischemia in choriocapillaris secondary to PDT may contribute to formation of CNV in chronic CSC with unhealthy RPE layers^21^. Thus, various ‘safety-enhanced’ PDT protocols have been devised to optimize treatment outcomes. They typically use reduced dose or reduced fluence^22^. However, secondary CNV could develop even after half dose PDT^23^.

In our series, 12 of 16 eyes in Group 1 underwent PDT or focal laser treatment. It is noteworthy that there was no significant difference in frequencies of application, parameters of PDT, or focal laser between Group 1 and Group 3. Despite the result, we cannot completely exclude the possibility that CNV may develop due to rupture of Bruch’s membrane by focal laser treatment or due to inner choroidal hypoxia induced by PDT.

Based on angiography, this study revealed that the major type of secondary CNV was the classic type (76.7%), rather than the occult type (23.3%). The angiography of secondary CNV was mainly characterized by classic type of focal dense leakage. Apparently, this finding is in contrast with previous reports that claimed a higher incidence of type 1 CNV than type 2 CNV in the eyes featuring CSC. Type 1 CNV has been known to commonly manifest on FA as an occult type of leakage. However, previous studies have shown that OCT scans in type 1 CNV are not often compatible with the occult type in angiography^24–26^. Chhablani *et al*.^18^ reported that the proportion of classic CNV was almost equal to that of occult CNV on OCT. Proportions can vary, however, depending on whether the study included polypoidal choroidal vasculopathy (PCV) or not. In addition, there have been reports of neovascularization under the flat irregular PED, i.e. pachychoroid neovasculopathy, in the eyes with pachychoroid features^9,20^. In those studies, the prevalence of the type 1 CNV should be high. However, those pachychoroid neovasculopathy and PCV cases should be discriminated from our study cases, where secondary CNV was noted after the resolution of SRF associated with CSC. On the other hand, it is interesting to note the hyperreflective vertical finger-like projections into the outer retina and the pitchfork sign^27^ in some CNVs secondary to CSC (as in Fig. 2E). The pitchfork sign has been noted exclusively in inflammatory CNV. Because uveitic cases were excluded in our study, however, further observation is required in this regard.

In our study, we collected cases of prior typical CSC with pachychoroid features without polypoidal structures or secondary CNV at baseline by multimodal imaging, such as SD-OCT, ICGA, and FA. PCV masquerading as CSC with signs of a branching vascular choroidal network or polypoidal lesions in ICGA images were carefully reviewed and excluded. The resolution of SRF after PDT or focal laser photocoagulation also supported the diagnosis of CSC at baseline. Furthermore, there were marked differences in angiography at baseline and at development of secondary CNV. Therefore, although we cannot completely exclude the possibility of masquerading CNV at the time of CSC diagnosis, the likelihood that it was present in our study is small. Although not applied in this study, OCT angiography is a useful tool to identify CNV in those eyes^5,6,28^.

We excluded cases where PCV developed after a prior history of CSC, as was done in the previous study^2^. This was done, in part, because the current study focused on investigating the risk factors and outcomes of secondary CNV, other than PCV. Interestingly, PCV was noted infrequently after the diagnosis of prior CSC. Only 2 eyes were excluded from this study due to development of PCV after an episode of CSC.

Double layer sign could present as a manifestation or precursor lesion of PCV^29,30^. This sign may reflect presence of fibrous tissue harbored by branching vascular network in PCV^30^. However, this sign is not pathognomonic of PCV. It can be found in CSC, and usually corresponded to thinning of inner choroid and pachyvessels^20,31,32^. Interestingly, approximately half of eyes in Group 1 manifested double layer sign at CSC diagnosis. This was significantly different, compared to that in Group 3 in which it was detected in 20% of eyes. Risk of secondary CNV was 12.3 times higher in eyes with double layer sign. The following may postulate reasons why risk of secondary CNV increases when there is double layer sign in eyes with CSC. First, double layer sign is associated with more extensive choroidal hyperpermeability. It may occur in more severe perturbation of RPE and in the inner choroidal layer. Therefore, it may increase risk of developing secondary CNV. Second, presence of double layer may impede transport of metabolite. As a result, outer retinal hypoxia could be exacerbated. This may increase risk of developing secondary CNV. Third, as mentioned above, double layer sign may harbor relatively inactive CNVs in dormant form^29,33^. Later, it may manifest active form of CNV. OCT angiography and other studies with imaging modalities will be helpful in clarifying these possibilities. Recently, an OCT angiographic study of chronic CSC revealed that CNV was detected in a third of eyes with flat irregular PED, which corresponded to the double-layer sign^33^. The neovascular activity was usually of low grade. Another study utilizing OCT angiography in pachychoroid spectrum disease supports the diagnostic value of the shallow irregular PED as a potential biomarker of type 1 neovascularization^32^.

This study also demonstrated that CSC accompanied by RPE changes revealed approximately 9 times higher risk for development of secondary CNV. Pigmentary changes in eyes with early AMD are a well-known risk factor for neovascular AMD. Derangements in RPE cells cause disruption of homeostasis in the outer retina and choriocapillaris. This may result in outer retinal hypoxia and promote incidence of secondary CNV.

Systemic hypertension is a risk factor of CSC in several studies, with odds being 1.7–3.3 times higher than that in the normal control group^34,35^. This study further disclosed that presence of systemic hypertension in CSC patients increased risk of secondary CNV by 7.7 times. Elevated systemic blood pressure and resultant arteriolosclerotic process may severely affect choroidal vessels, such as occlusion of choriocapillaris with subsequent breakdown of the outer blood-retinal barrier. These changes could lead to fluid leakage in the sub-RPE space and development of CSC. The hypoxic condition in choriocapillaris may lead to development of secondary CNV. Conversely, abnormal neovascularization in which renin-angiotensin-system plays a critical role may be involved in development of CNV in patients with systemic hypertension^36^.

Several treatments for CNV secondary to CSC have been reported, including PDT^11,14^ and surgical removal of CNV^37^. Recently, intravitreal injection of anti-angiogenics was shown to result in visual and anatomic improvements for CNV secondary to CSC^12,16–19,38–41^. Additionally, anti-VEGF injection, combined with reduced-fluence PDT was reported to be effective in managing secondary CNV in chronic CSC^42^. In our study, approximately two thirds of eyes treated with anti-VEGF alone achieved significant visual improvement and anatomically stable states, after only a limited number of injections. Anti-VEGF injection combined with PDT for secondary CNV also yielded viable therapeutic effects, supporting results of a previous study^42^.

This study has several limitations. Reflecting the rarity of developing CNV after CSC, this study was inherently limited by the retrospective nature of the study design. Thus, certain variabilities of imaging techniques, especially in OCT imaging, should be considered. In addition, statistical power was as low as 58% using proportion power analysis, due to the limited number of cases, although the sample size was relatively large compared to previous studies. Despite limitations, this study has several strengths. To our knowledge, it was the first study that compared CSC of control group and CSC of developing CNV secondarily. Additionally, most of our patients underwent ICGA, FA, and spectral domain OCT, enabling detailed analysis of features of CSC and secondary CNV.

In conclusion, this study identified hypertension, double layer sign, and RPE changes as independent risk factors for secondary CNV subsequent to CSC. Prompt and proper diagnosis, as well as anti-vascular endothelial growth factor treatment could achieve viable visual and anatomical prognoses. Therefore, surveillance and education for patients with these risk factors should be encouraged to facilitate early diagnosis and prompt treatment.

## Patients and Methods

Medical records of patients with CSC that visited retina clinics of Samsung Medical Center and Bundang Seoul National University Hospital November 1, 2002–December 31, 2016 were retrospectively reviewed. This study was approved by the Institutional Review Board of each hospital. Prior written informed consent for the use of medical records was obtained from all patients, and data collection followed tenets of the Declaration of Helsinki. We divided patients into three groups. Group 1 consisted of patients in whom CSC with SRF had been confirmed by ICGA, FA, and OCT, the resolution of SRF with or without treatment had also been confirmed during follow-up of those cases, and where secondary CNV was noted at some time later. Group 2 consisted of patients who were diagnosed with CNV in eyes secondary to putative CSC. We used the term “putative” because we didn’t see the patients previously, and thus, the presence of prior CSC with SRF had not been verified.

The diagnosis of secondary CNV was based on multimodal imaging, and included such features as^20,43^: (1) sub-retinal or sub-RPE hemorrhage, (2) sub-retinal turbid exudation, (3) evidence of fibrovascular PED, (4) ill-defined vascular leakage on FA, (5) abnormal vascular branching network on early phases of ICGA, and (6) late-staining plaque at the late phase of ICGA. Putative CSC was defined when the following features were found: (1) choroidal vascular dilation and hyperpermeability on ICGA, (2) RPE changes including atrophic tract on FA and OCT, (3) diffuse irregular hyperfluorescent spots on FA and ICGA, and (4) pachychoroid on enhanced depth imaging OCT. Pachychoroid was defined as an abnormal increase in the choroidal thickness above the upper quartiles found in the normal population^44^, and often manifested dilatation of the large outer oval choroidal vessels (Haller’s layer), compressing the overlying choriocapillaris and Sattler’s layer^6,10^.

Exclusion criteria included previous history of other retinal diseases such as diabetic retinopathy, age-related macular degeneration, and uveitis before diagnosis of CSC, or secondary CNV. Thus, patients with CSC should have no inflammatory cells in the anterior chamber or in the vitreous at the time of CSC diagnosis, and no other lesions outside the macula. Patients without angiography were excluded. Group 3 consisted of 250 randomly selected patients in whom a prior episode of CSC with SRF had been confirmed by ICGA, FA and OCT, and the resolution of SRF had also been confirmed during follow-up of those cases. Development of secondary CNV, however, was not noted during follow-up visits. Group 3 is regarded as a control group for Group 1.

### Ophthalmologic evaluation and imaging modalities

Each patient underwent comprehensive ophthalmologic examinations, including best corrected visual acuity and fundus examination. Color fundus photography, FA, and/or ICGA (Heidelberg Spectralis, Heidelberg, Germany) with OCT were obtained for each patient. OCT images were obtained with Cirrus SD-OCT (Carl Zeiss Meditec, Dublin, California, USA) and/or Spectralis SD-OCT (Heidelberg Engineering, Heidelberg, Germany). Enhanced depth imaging was used to evaluate choriocapillaris layer and choroidal thickness. One grader (G.I.L.) performed all measurements and evaluated the imaging features. When the analysis was ambiguous, the final decision was made after discussion with the supervising grader (S.W.K.).

Data regarding age at presentation, laterality, systemic diseases including diabetes mellitus and systemic hypertension, and treatment methods were retrieved. Regarding characteristics of CNV, presence of subretinal turbid exudation, subretinal hemorrhage on OCT and funduscopy, CNV location (subfoveal, juxtafoveal, and extrafoveal), CNV type (occult vs. classic type) on FA, and presence of hyperpermeability on ICGA were analyzed.

### Risk factor analysis

To identify local risk factors for development of CNV, we analyzed fundus findings of multimodal imaging at the time of CSC diagnosis in Group 1 prior to the onset of secondary neovascularization with Group 3; drusen, drusen-like deposits, and subretinal deposits on fundus color photography, PED, subretinal fibrin, ellipsoid zone disruption, RPE tear, pachyvessel, double layer sign, RPE change on fundus color photography and OCT, the number of hyperfluorescent spots (0,-no spot; 1,-less than 10 spots; 2, – more than 10 spots but less than 30 spots; 3,-more than 30 spots) and locations (including subfoveal, perifoveal, peripapillary, and other locations of retina) on mid-late phase of ICGA, choroidal hyperpermeability on early phase of ICGA, leakage type including ink blot, smoke-stack, and vague, and the number of leakage points on FA. Other features included RPE atrophy and RPE tracks on angiography. Drusen-like deposits were defined as sub-retinal or sub-retinal-pigment-epithelial grayish yellow deposits larger than 63 μm in size, with irregular, but discrete margins^45^. Subretinal fibrin was defined as hyperreflectivity in the subretinal space, corresponding to the fibrin material^46^, and subretinal turbid exudation was defined as fibrin leakage through the RPE defects, leading to photoreceptor layer defects^47,48^. Pachyvessel was defined as a dilatation of the large outer oval choroidal vessels (Haller’s layer), compressing the overlying choriocapillaris and Sattler’s layer^6^. The double-layer sign consisted of hyperreflective material between an undulated RPE line and a hyperreflective straight line, representing Bruch’s membrane^29^ on OCT, and was confirmed if it was angiographically silent.

Changes in logMAR best corrected visual acuity (BCVA) from initial visit to last follow-up, the proportion of eyes with stable state, number of injections, and duration until CNV regression were analyzed. Stable state was defined as a state not needing further treatment. CNV regression was defined as consolidation or complete resolution of subretinal fluid.

### Statistical analysis

Visual acuity at initial visit and the last follow-up was converted into logMAR for statistical analysis. Chi-square test was used to compare CNV type, location, and treatment response with stable state. Wilcoxon signed rank test was used to compare logMAR BCVA changes. Risk factors of secondary CNV were analyzed with univariate and multivariate logistic regression analyses. All statistical analysis was performed using SPSS software version 23.0 (SPSS Inc., Chicago, IL) and SAS software version 9.4 (SAS institute, Cary, NC, USA). A p-value of less than 0.05 was considered statistically significant.

## Supplementary information

Supplement table 1

## Data Availability

The datasets generated during and/or analyzed during the current study are available from the corresponding author on reasonable request.
