# Supplementary material for: Risk Factors and Outcomes of Choroidal Neovascularization Secondary to Central Serous Chorioretinopathy
Source: Sci Rep. 2019 Mar 8;9:3927. doi: 10.1038/s41598-019-40406-y (PMC6408555; doi:10.1038/s41598-019-40406-y)
Supplement: Supplementary file 1 — Supplement table 1 [file 41598_2019_40406_MOESM1_ESM.docx]

Title:

**Risk Factors and Outcomes of Choroidal Neovascularization Secondary to Central Serous Chorioretinopathy**

Name of authors:

Ga-In Lee^1^, A Young Kim^1^, Se Woong Kang^1^, Soo Chang Cho^2,3^, Kyu Hyung Park^2^, Sang Jin Kim^1^, Kyung Tae Kim^1^

Institutional affiliation:

*^1^Departments of Ophthalmology, Samsung Medical Center, Sungkyunkwan University School of Medicine, Seoul, Korea*.

*^2^Department of Ophthalmology, Seoul National University Bundang Hospital, Seoul National University College of Medicine, Seoul, Korea.*

*^3^Department of Ophthalmology, Kyungpook National University Hospital, Daegu, Republic of Korea.*

Address correspondence and reprint requests to

Se Woong Kang, MD, PhD

Department of Ophthalmology, Samsung Medical Center, Irwon-Ro 81, Gangnam-Gu, Seoul, South Korea, 06351

Tel: +82-2-3410-3562, Fax: +82-2-3410-0074

E-mail: [swkang@skku.edu](mailto:kangsewoong@gmail.com)

**Supplement Table 1**. Risk factors of CNV secondary to CSC, assessed by univariate analysis of baseline characteristics of Group1 and Group 3

| Parameter | Group 1, (N = 16) | Group 3, (N = 250) | P-value* |
| --- | --- | --- | --- |
| Diabetes mellitus, n/% | 2 (12.5) | 12 (4.8) | 0.21 |
| Systemic hypertension, n/% | 7 (43.8) | 48 (19.2) | 0.03 |
| Age (y) , n/% | 44.94±14.23 | 48.69±9.47 | 0.14 |
| Sex (male: female) | 11:5 | 195:55 | 0.40 |
| Focal laser, n/% | 4 (25.0) | 42 (16.8) | 0.41 |
| PDT, n/% | 12 (75.0) | 146 (58.4) | 0.20 |
| Macula edema, n/% | 1 (6.3) | 1 (0.4) | 0.046 |
| Pigment epithelial detachment, n/% | 1 (6.3) | 62 (24.8) | 0.14 |
| RPE tear, n/% | 0 (0.0) | 6 (2.4) | 0.99 |
| Subretinal fibrin, n/% | 6 (37.5) | 110 (44.0) | 0.76 |
| Ellipsoid zone disruption, n/% | 5 (31.2) | 24 (9.6) | 0.008 |
| Double layer sign, n/% | 8 (50.0) | 52 (20.8) | 0.007 |
| Locations of hyperfluorescent spots |  |  |  |
| Subfovea, n/% | 1 (6.2) | 70 (28.0) | 0.16 |
| Perifovea, n/% | 4 (25.0) | 187 (74.8) | 0.002 |
| Peripapilla, n/% | 4 (25.0) | 75 (30.0) | 0.86 |
| Rest of other area, n/% | 9 (56.2) | 143 (57.2) | 0.28 |
| Pachyvessels, n/% | 7 (43.8) | 168 (67.2) | 0.51 |
| Number of hyperfluorescent spots† (0/1/2/3) | 1/6/4/1 | 20/83/79/60 | 0.49 |
| Leakage pattern‡ (0/1/2/3) | 0/8/4/2 | 19/138/34/52 | 0.26 |
| Hyperpermeability, n/% | 13 (81.2) | 211 (84.4) | 0.99 |
| Leaking points, n/% | 1.43±0.85 | 1.05±0.50 | 0.01 |
| Descending atrophic tract, n/% | 1 (6.2) | 6 (2.4) | 0.32 |
| Drusen, n/% | 0 (0.0) | 12 (4.8) | 0.99 |
| Drusen-like deposits, n/% | 2 (12.5) | 55 (22.0) | 0.43 |
| RPE change, n/% | 12 (75.0) | 97 (38.8) | 0.004 |
| PDT = photodynamic therapy; RPE = retinal pigment epithelium.  *Statistical analysis with Univariate regression analysis.  †Number of hyperfluorescent spots; 0 – no spot, 1 – less than 10 spots, 2 – more than 10 spots and less than 30 spots, 3 – more than 30 spots.  Leakage pattern; 0 – no, 1 – ink blot pattern, 2 – smoke stack pattern, 3 – vague pattern. | | | |
